# An ancient whole-genome duplication event and its contribution to flavor compounds in the tea plant (*Camellia sinensis*)

**DOI:** 10.1038/s41438-021-00613-z

**Published:** 2021-08-01

**Authors:** Ya Wang, Fei Chen, Yuanchun Ma, Taikui Zhang, Pengchuan Sun, Meifang Lan, Fang Li, Wanping Fang

**Affiliations:** 1 College of Horticulture, Nanjing Agricultural University, Nanjing, 210095 China; 2 College of Life Sciences, Fudan University, Shanghai, 200433 China; 3 Key Laboratory of Bio-Resource and Eco-Environment of Ministry of Education, College of Life Sciences, Sichuan University, Chengdu, 610065 China; 4 College of Life Sciences, North China University of Science and Technology, Tangshan, 063099 China

**Keywords:** Population genetics, Evolution

## Abstract

Tea, coffee, and cocoa are the three most popular nonalcoholic beverages in the world and have extremely high economic and cultural value. The genomes of four tea plant varieties have recently been sequenced, but there is some debate regarding the characterization of a whole-genome duplication (WGD) event in tea plants. Whether the WGD in the tea plant is shared with other plants in order Ericales and how it contributed to tea plant evolution remained unanswered. Here we re-analyzed the tea plant genome and provided evidence that tea experienced only WGD event after the core-eudicot whole-genome triplication (WGT) event. This WGD was shared by the Polemonioids-Primuloids-Core Ericales (PPC) sections, encompassing at least 17 families in the order Ericales. In addition, our study identified eight pairs of duplicated genes in the catechins biosynthesis pathway, four pairs of duplicated genes in the theanine biosynthesis pathway, and one pair of genes in the caffeine biosynthesis pathway, which were expanded and retained following this WGD. Nearly all these gene pairs were expressed in tea plants, implying the contribution of the WGD. This study shows that in addition to the role of the recent tandem gene duplication in the accumulation of tea flavor-related genes, the WGD may have been another main factor driving the evolution of tea flavor.

## Introduction

Tea beverages made from tea plants (*Camellia sinensis*) are known as the world’s oldest (~3000 BC) and most popular nonalcoholic caffeinated beverages. Consumed by more than 3 billion people in more than 160 countries^1^, tea beverages have high economic and cultural value. With the rapid development of genome sequencing technologies^2,3^, a number of genomes of *C. sinensis* have been obtained, including those of *C. sinensis* var. *sinensis* (CSS) cv. “Shuchazao,”^4–6^ cv. ‘“Biyun,”^7^ cv. “Longjin43,”^8^ and the wild variety “DASZ”^9^ and *C. sinensis* var. *assamica* (CSA) cv. “Yunkang 10”^10^. These genomic data and results greatly accelerated research in tea plant science^11^.

The molecular mechanisms underlying tea flavor have been widely explored^11,12^. The metabolic pathways and accumulation mechanisms of catechins, caffeine, and theanine, the main secondary metabolites in tea, have been studied thoroughly^11^. The question of how tea plants evolved to accumulate specialized secondary metabolites has long attracted scientists. Tandem duplication has long been considered a key mechanism by which plants expand their accumulation of secondary metabolites. For example, the genes in the biosynthetic pathway of caffeine^13^ in tea plants and the biosynthetic pathway of morphine in poppy^14^ expanded in a similar way, through massive tandem gene duplication events. In addition, whole-genome duplication (WGD) is also considered an important factor leading to the development of stress resistance in plants^15,16^ and studies have also indicated that WGD contributed to the biosynthesis of secondary metabolites^17^. Due to recursive genome duplications and the loss of a large number of genes, large-scale gene relocations, the rearrangement of chromosome fragments, and fusion following genome duplication^18–22^, it is difficult to explore the evolutionary processes of plant genome duplication events and many researchers have developed tools to address this problem^23^. Therefore, how many rounds of WGD events tea plants experienced and what are their contributions to the evolution of secondary metabolites in tea plants were new questions that remain unanswered.

**Fig. 1:**
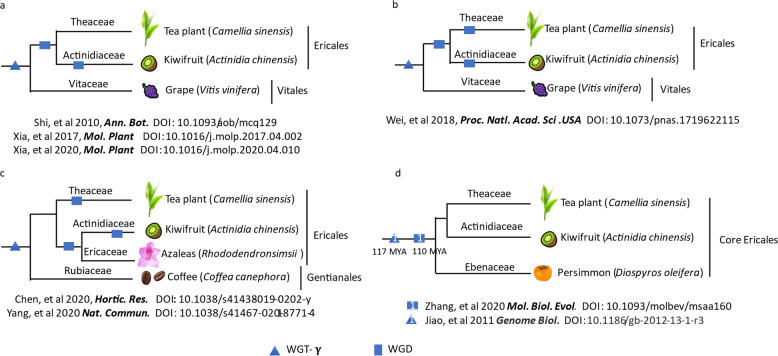
Current debates regarding whole-genome duplication in tea plants. **a** The tea plant experienced only one WGD after the WGT-γ. **b** The tea plant experienced two WGD events after the WGT-γ, one shared with kiwifruit (WGD-β, ~90–100 MYA), and one specific to tea plants (WGD-α, ~30–40 MYA). **c** The tea plant experienced only one WGD event after the WGT-γ and the WGD was tea plant specific. **d** Zhang et al. reported that the WGD occurred at ~100 MYA. The WGT-γ was reported to occur ~117 MYA

At present, the tea plant research community has obtained several genomes, but no unified conclusion regarding a WGD event in tea plants has been reached (Fig. 1). In 2010, Shi et al.^24^ suggested that tea and kiwifruit shared a WGD. Later studies of the CSA cv. “Yunkang 10”^10^ and CSS cv. “Shuchazao”^6^, as well as a study of widely cultivated azaleas (*Rhododendron simsii*)^25^ confirmed that tea plants experienced only one WGD event, which was shared with kiwifruit or rhododendron, after core-eudicot whole-genome triplication-γ (WGT-γ) (Fig. 1a). However, a genomic study of CSS cv. “Shuchazao”^4^ showed that the tea plant experienced two WGD events (Fig. 1b) after the WGT-γ: one was shared with kiwifruit (WGD-β, ~90–100 MYA) and one occurred in tea plants (WGD-α, 30–40 MYA). Chen et al.^5^ also studied cv. “Shuchazao” and found that tea plants experienced only one tea plant-specific WGD event following WGT-γ; this is the same as the conclusion of Yang et al.^25^ (Fig. 1c). In addition, the occurrence time of this WGD in order Ericales is still controversial; estimates include ~110 MYA by Zhang et al.^26^ (Fig. 1d)^26,27^ and ~50–57 MYA by Wang et al.^17^.

To this end, we re-analyzed the tea plant genomes and the genomes of related representative species. WGDs in rhododendron (*R. simsii*), kiwifruit (*Actinidia chinensis*) and persimmon (*Diospyros lotus*) (all belonging to order Ericales) have been reported^24,25,28–30^. In addition, the grape (*Vitis vinifera*) genome is relatively simple, experiencing only the core eudicot-specific WGT^31^. The genomes of rhododendron, kiwifruit, persimmon, and grape were used as references to reveal the WGD event in tea and to further explore the contribution of the WGD to the important secondary metabolites accumulated in tea plants.

## Results

### Evidence of an ancient WGD in the tea plant genome

**Fig. 2:**
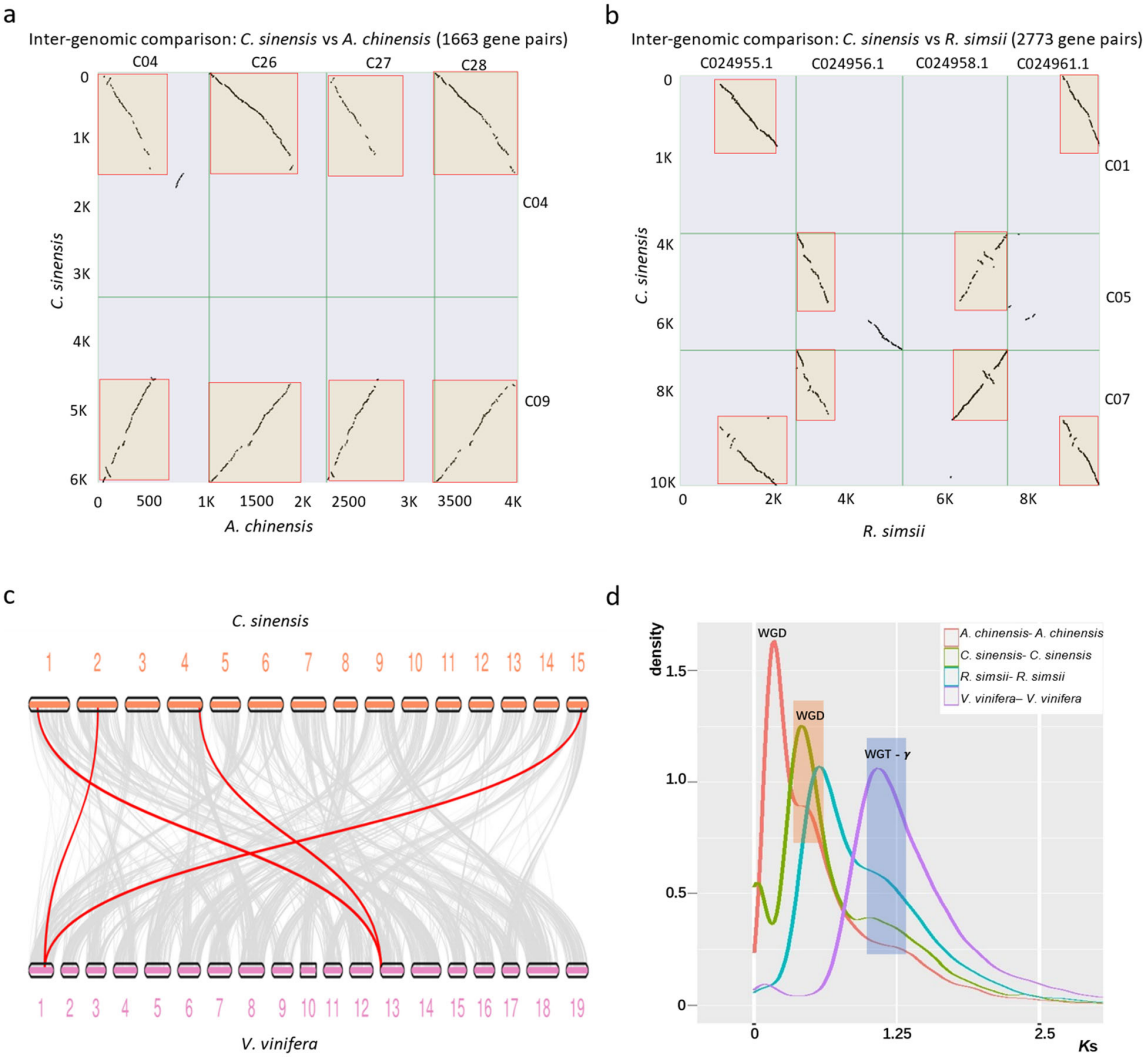
Syntenic relationships and the *K*s distribution suggested an ancient WGD event in the tea plant genome. **a** Homologous dot plot between certain tea plant (*C. sinensis*) and kiwifruit (*A. chinensis*) chromosomes. **b** Homologous dot plot between certain tea plant and rhododendron (*R. simsii*) chromosomes. **c** Syntenic relationships of tea plant and grape chromosomes. The blocks with syntenic relationships are connected by gray lines and red lines represent two examples in which a block of grape (*V. vinifera*) has syntenic relationships with two blocks of tea plant. **d** The distribution of *K*s for WGD gene pairs from kiwifruit, tea plant, rhododendron, and grape. The horizontal coordinates are *K*s and the ordinates indicate the density of the gene pairs corresponding to *K*s

We first studied the genomic syntenic relationships among the five species, i.e., tea plant, kiwifruit, grape, persimmon, and rhododendron (Supplementary Fig. S1). With a total of 1342 syntenic blocks covering 21,873 gene anchor pairs (Supplementary Fig. S1e), the syntenic blocks between tea plant and kiwifruit showed a 2 : 4 syntenic relationship. For example, CsChr04 and CsChr09 in tea plant matched AcChr04, AcChr26, AcChr27, and AcChr28 in kiwifruit (Fig. 2a). In addition, a total of 849 syntenic blocks covering 16,827 homologous gene anchor pairs were detected between tea plant and rhododendron, showing a significant 2 : 2 syntenic relationship (Supplementary Fig. S1d). For example, CsChr01, CsChr05, and CsChr07 in tea plant matched C024955.1, C024956.1, C024958.1, and C024961.1 in rhododendron (Fig. 2b). Tea plants and grapes showed 2 : 1 syntenic relationships (Fig. 2c), and the same relationship was found in the analysis of wild tea plants DASZ and grapes (Supplementary Fig. S1g, h). These results indicated that tea plants experienced WGD after the WGT.

The distribution of the *K*s of the syntenic paralogs of tea plant, kiwifruit, grape, and rhododendron also revealed a similar result (Fig. 2d). We constructed the *K*s distributions for the tea plant, rhododendron, persimmon, kiwifruit, and grape (Fig. 2d and Supplementary Fig. S4). The distribution of *K*s showed that tea plant had a *K*s peak at 0.425, which is on the left side of the grape peak at 1.088, suggesting that tea experienced an additional WGD after the WGT-γ. Rhododendron had a *K*s peak similar to that in tea plants. Only kiwifruit showed two peaks, indicating that kiwifruit experienced additional WGDs that were not experienced by tea plant or rhododendron.

### Molecular dating of this ancient WGD

**Fig. 3:**
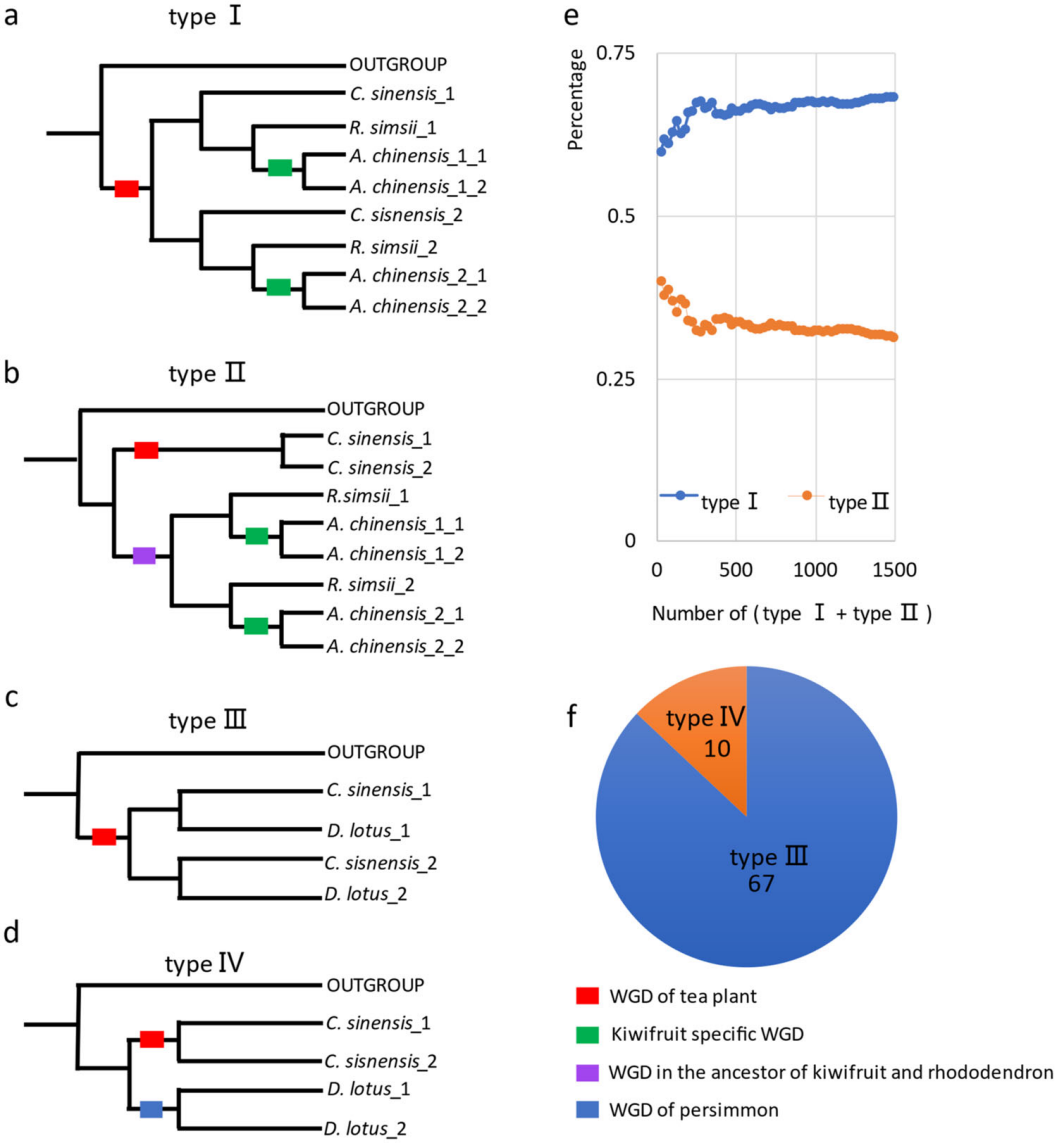
Phylogenetic trees of syntenic genes showing that the WGD was shared by tea plant, kiwifruit, rhododendron, and persimmon. **a** Phylogenetic tree indicating that the tea plant (*C. sinensis*) shared the WGD with kiwifruit (*A. chinensis*) and rhododendron (*R. simsii*) (type I). **b** Phylogenetic tree indicating that the tea plant (*C. sinensis*) experienced the WGD independently (type II). **c** Phylogenetic tree indicating that persimmon (*D. lotus*) shared the WGD with tea plant (*C. sinensis*) (type III). **d** Phylogenetic tree indicating that persimmon (*D. lotus*) did not share the WGD with tea plant (*C. sinensis*) (type IV). **e** Statistics on the proportions of two types of phylogenetic trees (type I and type II). **f** Statistics on the proportions of two types of phylogenetic trees (type III and type IV)

**Fig. 4:**
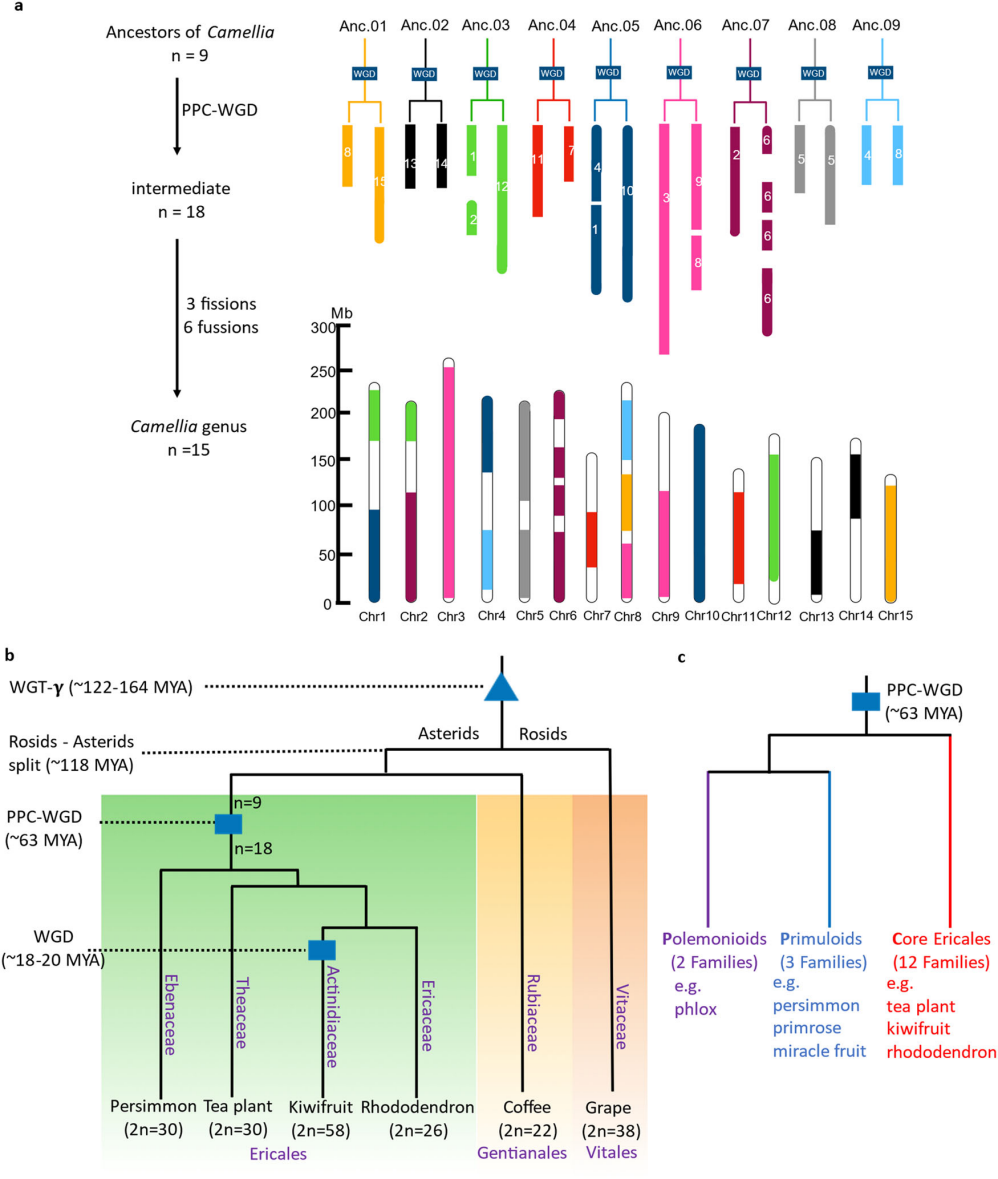
The PPC-WGD shared by four families within the order Ericales and the evolution of tea plant chromosomes. **a** Evolutionary patterns of tea plant chromosomes after WGD. **b** The species tree of tea plant and related species. The WGT and WGD are labeled triangles and rectangles, respectively. **c** Polemonioids, Primuloids, and core Ericales shared the PPC-WGD

We have shown that tea plants experienced only one WGD after the WGT-γ. However, was this WGD tea plant specific or shared with kiwifruit, or even more species? It cannot be clearly determined through only the *K*s distribution shown in Fig. 2d. Therefore, we constructed phylogenetic trees to determine whether this WGD is tea plant specific. We finally constructed 2798 single-copy nuclear gene-based phylogenetic trees covering tea plant (order Ericales, family Theaceae), rhododendron (order Ericales, family Ericaceae), kiwifruit (order Ericales, family Actinidiaceae), and outgroups (grape or coffee) (see “Methods” and Supplementary Table S2). Among them, type I (supporting tea plant, rhododendron, and kiwifruit sharing the WGD) (Fig. 3a) had 1021 trees, accounting for 36.5% of all phylogenetic trees. Type II (supporting tea plant experiencing the WGD independently) (Fig. 3b) had 471 trees, accounting for 16.8% of all trees. The rest of the trees were types other than type I or type II. We determined the proportions of the two types (Fig. 3e). The value of type I/(type I + type II) was 68.5%, whereas type II/(type I + type II) was 31.8%; the proportion of type I trees was more than twice that of type II trees. Another question was whether the close relative persimmon (*D. lotus*, order Ericales, family Ebenaceae) shared this WGD or not. A total of 168 single-copy nuclear gene-based phylogenetic trees covering tea plant, persimmon, and outgroups (grape or coffee) were constructed (Supplementary Table S2). Among them, 67 (40%) phylogenetic trees (type III) (Fig. 3c) clearly supported that tea plants and persimmons shared the WGD. Only ten (6%) phylogenetic trees (type IV) (Fig. 3d) supported that the two species did not share the WGD (Fig. 3f). Therefore, this evidence showed that the tea plant shared this WGD with rhododendron (family Ericaceae), kiwifruit (family Actinidiaceae), and persimmon (family Ebenaceae). According to the phylogenetic taxonomy of the order Ericales^26^, this WGD was shared by at least 17 families in three sections: Polemonioids, Primuloids, and core Ericales (PPC). Hence, this WGD was termed PPC-WGD in this study (Fig. 4b).

In addition, we inferred chromosome evolution in tea plants. The chromosomes were plotted before and after PPC-WGD based on the syntenic gene blocks within the tea plant genome (Supplementary Fig. S8). Prior to PPC-WGD, nine ancestral chromosomes (2*n* = 18) were reconstructed. PPC-WGD then produced 18 ancestral chromosome intermediates (2*n* = 36). After three fissions and six fusions, the number of tea plant chromosomes reached the current number, 15 (2*n* = 30) (Fig. 4a). The evolution of tea plant chromosomes from 7 chromosomes (prior to WGT-γ) to 9 chromosomes showed that almost all chromosomes were formed by a series of fusions, interchanges, and insertions (Supplementary Fig. S6).

To infer the occurrence time of the PPC-WGD, the *K*s = *t*/2*r* method, which has been widely applied to calculate the occurrence time of WGDs in many articles^25,28,32^, was used. The results showed that the time of the PPC-WGD was ~63 MYA (Fig. 4b), which is very close to the mass extinction at ~66 MYA at the Cretaceous-Paleogene (K-Pg) boundary^33^.

### Contributions of PPC-WGD to characteristic secondary metabolites in tea

**Fig. 5:**
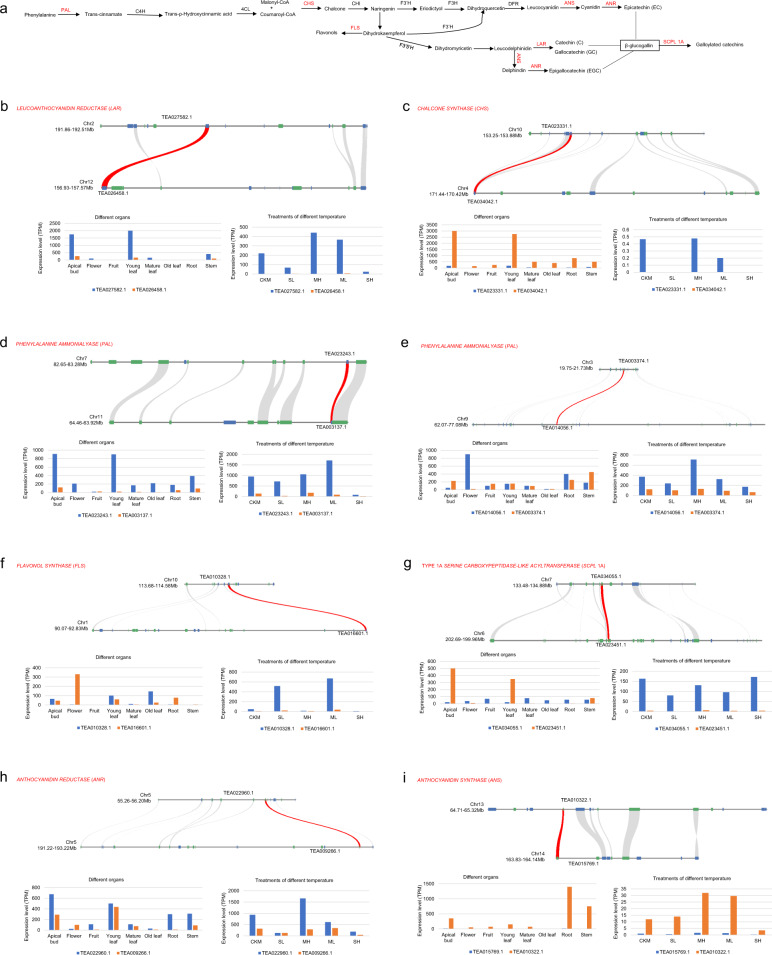
PPC-WGD contributed to the expansion of genes involved in the biosynthesis of catechins in tea plants. **a** The main biosynthesis pathways of catechins in tea plants. **b**–**i** Microsynteny visualization and expression chart of *LAR*, *PAL*, *CHS*, *FLS*, *SCPL*, *ANR*, and *ANS* genes in the biosynthesis pathway of catechins. CKM, SL, MH, ML, and SH represent CK, severe low temperature, moderate heat, moderate low temperature, and severe heat, respectively

Caffeine, theanine, and catechins are the three most characteristic secondary metabolites in tea, playing important roles in creating tea flavor. At present, a series of studies have revealed that tandem duplication is the main reason for the accumulation of these special secondary metabolites, such as caffeine^10^ and catechins^4^, in tea plants. However, whether the PPC-WGD contributed to the amplification of genes related to these special secondary metabolites in tea plants is unclear. The biosynthesis of catechins involves regulation by many key enzymes, including phenylalanine ammonia lyase (PAL), leucoanthocyanidin reductase (LAR), anthocyanidin reductase (ANR), and many other key enzymes^34^ (Fig. 5a). Our analyses showed that a pair of *LAR* genes (Fig. 5b), a pair of *CHALCONE SYNTHASE* genes (Fig. 5c), two pairs of *PAL* genes (Fig. 5d, e), a pair of *FLAVONOL SYNTHASE* (*FLS*) genes (Fig. 5f), a pair of *type 1A SERINE CARBOXYPEPIDA-LIKELTRANSFERASE* (*SCPL 1A*) genes (Fig. 5g), a pair of *ANR* genes (Fig. 5h), and a pair of *ANTHOCYANIDIN SYNTHASE* (*ANS*) genes (Fig. 5i) were gene pairs with strong syntenic relationships produced by the PPC-WGD. The expression profile of the duplicated genes showed that most of these genes had at least one copy and sometimes both copies had high expression in the apical bud and leaf organ. In addition, only one copy of *LAR* (*TEA026458.1*), *PAL* (*TEA003137.1*), and *ANS* (*TEA015769.1*) had low expression in plant organs; under the different temperature treatments, both copies of most genes showed high expression in plant organs or under the temperature treatments. For example, two copies of *ANR* were highly expressed in apical buds and young leaves (Fig. 5h). The *FLS* copy *TEA016601.1* had high expression in flowers, whereas *TEA0010328.1* had high expression in the third mature leaf at severe low temperature and moderate low temperature (Fig. 5f). Although the two copies of most PPC-WGD gene pairs did not have high expression in the same organs or the same temperature treatments, the two copies were expressed at higher levels in different organs or under different treatments, showing that the two PPC-WGD copies contribute to the biosynthesis of catechins in different organs and under different temperatures in tea plants.

**Fig. 6:**
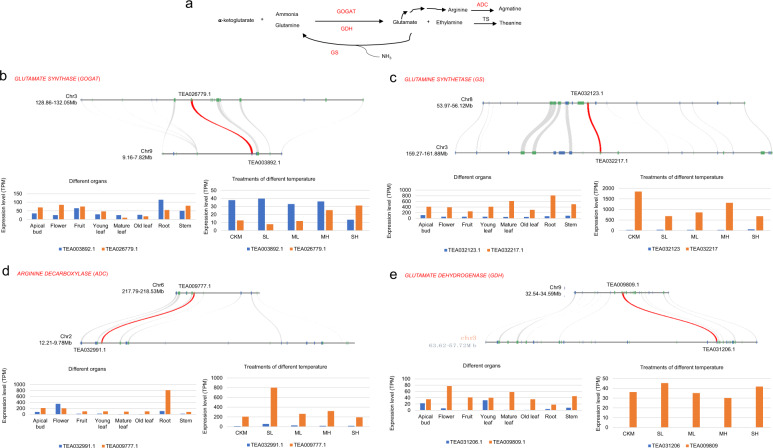
PPC-WGD contributed to the expansion of genes involved in the biosynthesis of theanine in tea plants. **a** The main biosynthesis pathways of theanine in tea plants. **b** Microsynteny visualization and expression chart of *GOGAT* genes in the biosynthesis pathway of theanine. **c** Microsynteny visualization and expression chart of *GS* genes in the biosynthesis pathway of theanine. **d** Microsynteny visualization and expression chart of *ADC* genes in the biosynthesis pathway of theanine. **e** Microsynteny visualization and expression chart of *GDH* genes in the biosynthesis pathway of theanine

Theanine, a nonprotein amino acid found in *Camellia* plants that accounts for ~70% of the total free amino acids in the new shoots of tea plants, is closely correlated with tea quality^35^. The main route for theanine biosynthesis progresses from glutamine to theanine^36^, including catalytic enzymes such as glutamate synthase (GOGAT), glutamine synthetase (GS), arginine decarboxylase (ADC), glutamate dehydrogenase (GDH), and theanine synthase (Fig. 6a). Our analysis showed that a pair of *GOGAT* genes, a pair of *GS* genes, a pair of *ADC* genes, and a pair of *GDH* genes in tea plant are anchor pairs duplicated by the PPC-WGD (Fig. 6). Expressional analyses showed that the two copies of *GOGAT* had high expression, both in different organs and under different temperature treatments. Other duplicates had high expression in tea plant organs or under different temperature treatments (Fig. 6). Together, these results suggested that PPC-WGD probably contributed greatly to the development of theanine biosynthesis.

**Fig. 7:**
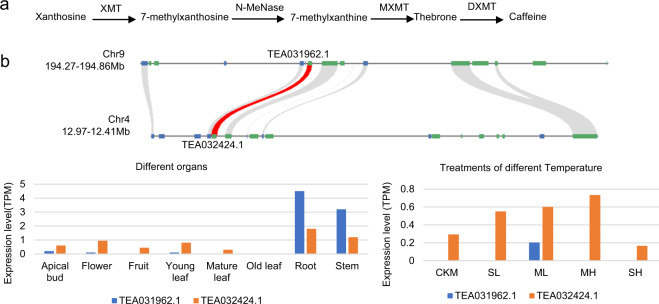
PPC-WGD contributed to the expansion of genes involved in the biosynthesis of caffeine in tea plants. **a** The main biosynthesis pathways of caffeine in tea plants. **b** Microsynteny visualization and expression of *NMT* genes in the biosynthesis pathway of caffeine

Caffeine (1,3,7-trimethylxanthine), a common ingredient found in tea, coffee, and cocoa, is an important flavor substance in tea that has many benefits for human health^37^. The main steps of caffeine biosynthesis involve three methylation steps from xanthosine to caffeine^38,39^. The tea plant pathway from xanthosine nucleosides to caffeine mainly depends on a continuum of three *N*-methyltransferases (NMTs), including xanthosine methyltransferase, 7-methylxanthine methyltransferase, and 3,7-dimethylxanthine methyltransferase (Fig. 7a). Our analyses showed that a pair of *NMT* genes in the tea plant was duplicated through the PPC-WGD (Fig. 7b). The expression profiles of the two duplicates showed relatively low expression in all tea plant organs, but this could be due to the specific spatial and temporal expression patterns or the induced expression of *NMT* genes under specific circumstances (Fig. 7b).

We then compared the gene numbers from those pathways, i.e., the catechins, theanine, and caffeine biosynthesis pathways, Supplementary Table S1) in persimmon and rhododendron. Only a few or even no homologous genes were found in rhododendron and persimmon, Supplementary Table S3), showing that although rhododendron and persimmon shared this PPC-WGD with tea plant, the tea plant was better able to the genes that participate in these pathways.

## Discussion

### Why PPC-WGD identification in tea plants has consistently been controversial

Explorations of the PPC-WGD in tea plants in different studies have reached inconsistent conclusions, probably for the following reasons. First, the PPC-WGD is relatively old (occurring more than 60 MYA) and lateral genome shuffling makes it even more difficult to identify. Second, the split between tea and other species in the Ericales almost followed the occurrence of the PPC-WGD, thus making it difficult to determine whether the PPC-WGD occurred before or within the tea plant split. Third, the highly random error rate of third-generation sequencing platforms will lead to bias in *K*s distributions. Fourth, the rates of gene retention and gene loss can be very different among different species; this may also be one of the reasons why it is difficult to identify whether species experienced the PPC-WGD together. Therefore, our combined method relying on a syntenic approach, a phylogenetic approach, and the *K*s distribution of anchor pairs allows us to comprehensively and accurately identify the PPC-WGD.

### The timing of the PPC-WGD

If this PPC-WGD is shared by tea plant, kiwifruit, persimmon, and rhododendron, as proposed by the phylogenetic relationship reported by Zhang et al.^26^, it is shared by at least 17 families in the order Ericales (Ericaceae, Cyrillaceae, Clethraceae, Actinidiaceae, Roridulaceae, Sarraceniaceae, Styracaceae, Diapensiaceae, Symplocaceae, Theaceae, Sladeniaceae, Pentaphylaceae, Ebenaceae, Primulaceae, Sapotaceae, Polemoniaceae, and Fouquieriaceae). However, to determine whether other families in the order Ericales also shared this PPC-WGD, more genomic data will be needed in the future.

In the results, we found that the PPC-WGD of tea plants occurred ~63 MYA, almost coinciding with the mass extinction at the K-Pg boundary. The K-Pg mass extinction is an significant event in the history of the earth^40,41^. A series of monocots and eudicots experienced WGDs at the K-Pg boundary and retained stress-related genes^15^. Many PPC-WGD genes related to stress resistance were also retained in tea plants (Supplementary Fig. S7).

### PPC-WGD and subsequent shuffling in the evolution of tea plant chromosomes

How did the chromosomes of tea plant (2*n* = 30) evolve? Previous genomic reports on tea plants^4–8^ and plants in the order Ericales^17,24,25,28–30,42,43^ did not provide any information on this question. To fill this gap, our study provides multiple lines of evidence to reveal the evolutionary history of tea plant chromosomes. Relying on genome synteny and comparative genomics, we showed that the tea plant has an ancestral chromosome base of 9 chromosomes (2*n* = 18). Then, the PPC-WGD produced 18 chromosomes, followed by 3 fissions and 6 fusions, and the extant 15 chromosomes (2*n* = 30) of most *Camellia* species were formed. In addition, we provide strong evidence that at least four families (Theaceae, Ericaceae, Actinidiaceae, and Ebenaceae) shared this PPC-WGD. Considering that there are 17 families in the PPC section in order Ericales, we believe that the number of ancestral chromosomal bases of the PPC section species is 9. Therefore, this study provides details about the chromosomal evolution of many important species, including tea plant, kiwifruit, rhododendron, and persimmon.

### PPC-WGD contributes to tea flavor

Caffeine, catechins, and theanine are responsible for the unique flavor of tea. Most previous studies have confirmed that the genes in the biosynthetic pathways of these secondary metabolites were expanded by tandem duplications. For example, in coffee, scientists revealed that *NMT* genes expanded through sequential tandem duplications^13^. Subsequent studies in tea plant also identified the *NMT* genes responsible for caffeine biosynthesis and the *SCPL* genes responsible for catechins biosynthesis, which were expanded by tandem duplications in the tea plant genome^4,10^.

Coffee, tea plant, and cocoa all belong to the core eudicots that experienced a γ-WGT at ~100 MYA. Coffee and tea plant are asterids, whereas cocoa is a rosid. Cocoa and coffee did not experience the lateral WGD, but the tea plant experienced the PPC-WGD, as we have shown with multiple lines of evidence. Coffee and cocoa did not experience the lateral WGD; thus, the accumulation of the three main secondary metabolites (caffeine, catechins, and theanine) could only occur due to segmental gene duplication or tandem gene duplications. In the tea plant, we found that multiple key genes expanded and were retained after the PPC-WGD, including eight pairs of genes associated with catechins, four pairs of key genes related to the biosynthesis of theanine, and a pair of *NMT* genes associated with the biosynthesis of caffeine. Compared to coffee and cocoa, tea generally had more paralogs of these genes (Supplementary Fig. S5). In addition, we showed that these genes were expressed in different organs, suggesting that the gene dosage contributed to the accumulation of secondary metabolites in the tea plant.

However, why did other species that experienced the PPC-WGD with tea plants, such as rhododendron, kiwifruit, and persimmon, not begin to accumulate these characteristic secondary metabolites? First, although tea plants shared the PPC-WGD with other plants in the order Ericales, tea plants better retained the relevant genes after the PPC-WGD. The retained PPC-WGD genes related to these pathways for characteristic secondary metabolites are far more abundant in tea than in rhododendron and persimmon (Supplementary Table S3), which indicates that tea better retained the genes that participate in these pathways. Second, due to the long-term differentiation and independent evolution of these species, there is no caffeine or theanine in rhododendron, kiwifruit, or persimmon^35,37^, indicating that there is no biosynthetic pathway responsible for caffeine or theanine in these species. Therefore, the retained PPC-WGD genes in these plants are very likely to perform different functions than those in tea plants.

## Experimental procedures

### Data sources

The coding sequence (cds) and generic feature format (gff) files, and the genomic data for the tea plant (CSS) were downloaded from GitHub (the analyses of the evolution of tea plant chromosomes and the contribution of PPC-WGD to tea flavor were based on this genome): https://github.com/JiedanChen/TeaGenomeData, TPIA^44^ (except for special annotations, other analyses were based on this genome): http://tpia.teaplant.org/index.html, and figshare^45^ (genome of wild tea plants DASZ): https://figshare.com. The cds and gff files and the genome data for grape (*V. vinifera*) were downloaded from Phytozome^46^: https://phytozome.jgi.doe.gov/pz/portal.html. The cds and gff files and the genome data for kiwifruit (*A. chinensis*) were downloaded from the Kiwifruit Genome Database^43^: http://kiwifruitgenome.org/organism/5. The cds and gff files and the genome data for rhododendron (*R. simsii*) were downloaded from NCBI: https://www.ncbi.nlm.nih.gov/. The cds and gff files, and the genome data for coffee (*Coffea canephora*) were downloaded from the Coffee Genome Hub^47^: http://www.coffee-genome.org/. The cds and gff files, and the genome data for persimmon (*D. lotus*) were downloaded from Persimmon DB: http://persimmon.kazusa.or.jp/. The relevant genes identified in the tea plant pathways for catechins, caffeine, and theanine biosynthesis were derived from previously reported results^4^, and the relevant gene expression data for the different organs were derived from TPIA^44^: http://tpia.teaplant.org. We cultivated tea seedlings at different temperatures to obtain transcriptome data at different temperatures.

### Methods

To identify the homologous gene pairs and syntenic relationships between tea and other species, we used MCscan^48^ (https://github.com/tanghaibao/jcvi/wiki/MCscan-(Python-version)) to perform interspecies syntenic analysis, to obtain homologous gene blocks and gene pairs between species, as well as syntenic plots of homologous gene pairs. To characterize the synonymous substitution rates (*K*s) for homologous genes within species, we used DupGen_finder^49^ to identify the WGD gene pairs within species and then used KaKs_Calculator (2.0)^50^ to calculate the *K*s values of those gene pairs within species and homologous gene pairs between species obtained by MCscan based on the NG model. We made a preliminary *K*s density map for each species (Supplementary Fig. S2). In theory, the divergence time of the three species (tea plant, rhododendron, and kiwifruit) and grape should be consistent, so the divergence peak of *K*s (Supplementary Fig. S4) of the three species and grape should also be consistent. Therefore, we calculated the correction coefficient of kiwifruit (*C*_ac_) and rhododendron (*C*_rs_) using the *K*s peak of tea and grape (*K*s_css-vv_) as a reference: *K*s_css-vv_ = *K*s_ac-vv_ * *C*_ac_ = *K*s_rs-vv_ * *C*_rs_. The original *K*s (Supplementary Fig. S3) of kiwifruit and rhododendron were then corrected with *C*_ac_ and *C*_rs,_ respectively. Finally, we used R to plot the bar chart or curve chart of the *K*s values based on the same parameters, such as bins.

To determine whether the WGD event in tea was shared with species such as rhododendron and kiwifruit, we first used DupGen_finder^49^ to determine the WGD gene pairs for tea plant, kiwifruit, and rhododendron. We then selected the syntenic genes of coffee or grape as outgroups and identified the syntenic genes of kiwifruit and rhododendron. After the selection of the appropriate genes, we used Mafft^51^ for multisequence comparison with the -auto parameter option. Then, the comparison sequences were compared with FastTree^52^ to construct phylogenetic trees with default parameters. Finally, MEGAX^53^ was used to view and adjust the tree files. Then, we constructed phylogenetic trees for the persimmon and tea plant sequences in type I (Fig. 3a).

We used the *K*s = *t*/2*r* method, which has been widely used in molecular dating^25,28,32^, to calculate the WGD occurrence time. We calculated the *r*-value based on the divergence time of tea plant and grape (the rosid-asterid split) at ~118 MYA^32^ and the *K*s value (0.781) corresponding to the split peak of the tea plant and grape (Supplementary Fig. S4). Then, we calculated the time of the WGD based on the *r*-value and the *K*s value (0.425) corresponding to the tea plant *K*s peak (Supplementary Fig. S3).

We inferred the number of tea plant chromosomes before the WGD and their evolution after the WGD based on the homologous gene blocks within the tea plant genome (Supplementary Fig. S8). Large fragments of syntenic blocks are thought to have been doubled by the same chromosome fragment, which suggested the evolution of the tea plant chromosomes.

To explore whether the WGD contributed to the development of important secondary metabolites in tea plant, we first looked for the genes responsible for the biosynthesis pathways of important secondary metabolites in tea plant. Then, we searched these genes as anchor pairs by checking the MCscan-generated syntenic genes (Supplementary Table S1). The gene expression data for different organs were obtained from the TPIA^44^ database. We used fastp^54^ to preprocess the raw fastq data, STAR^55^ for sequence matching, and RSEM^56^ to calculate the amount of expression, to obtain expression data for the tea plants at different temperatures.

AgriGO v2.0^57^ was used to conduct Gene Ontology analyses. We conducted a protein BLAST search to identify the homologous genes for the genes mentioned in those pathways, i.e., the catechins, theanine, and caffeine pathways (Supplementary Table S1), in persimmon and rhododendron, which were retained in the WGD. We performed a BLAST search with the pep sequence of the retained WGD genes of tea plant, persimmon, and rhododendron, and the pep sequences of the genes in Supplementary Table S1 were used as the query.

## Supplementary information

Supplementary data
