# Supplementary material for: An ancient whole-genome duplication event and its contribution to flavor compounds in the tea plant (Camellia sinensis)
Source: Hortic Res. 2021 Aug 1;8:176. doi: 10.1038/s41438-021-00613-z (PMC8325681; doi:10.1038/s41438-021-00613-z)
Supplement: Supplementary file 1 — Supplementary data [file 41438_2021_613_MOESM1_ESM.docx]

**Supplementary data**

**Supplemental Table 1.** The retained WGD that play an important role in the synthesis pathway of theanine, catechins and caffeine.

| **Pathway** | **Gene** | **Gene pair ID** | |
| --- | --- | --- | --- |
| caffeine | *NMT (N-METHYLTRANSFERASE)* | TEA031962.1 | TEA032424.1 |
| catechins | *SCPL 1A (TYPE 1A SERINE CARBOXYPEPTIDASE-LIKE ACYLTRANSFERASE)* | TEA034055.1 | TEA023451.1 |
|  | *PAL (PHENYLALANINE AMMONALYASE)* | TEA014056.1 | TEA003374.1 |
|  |  | TEA023243.1 | TEA003137.1 |
|  | *ANR (ANTHOCYANIDIN REDUCTASE)* | TEA022960.1 | TEA009266.1 |
|  | *ANS (ANTHOCYANIDIN SYNTHASE)* | TEA015769.1 | TEA010322.1 |
|  | *LAR (LEUCOANTHOCYANIDIN REDUCTASE)* | TEA027582.1 | TEA026458.1 |
|  | *FLS (FLAVONOL SYNTHASE)* | TEA010328.1 | TEA016601.1 |
|  | *CHS (CHALCONE SYNTHASE)* | TEA023331.1 | TEA034042.1 |
| theanine | *GOGAT (GLUTAMATE SYNTHASE)* | TEA003892.1 | TEA026779.1 |
|  | *GS (GLUTAMINE SYNTHETASE)* | TEA032123.1 | TEA032217.1 |
|  | *ADC (ARGININE DECARBOXYLASE)* | TEA032991.1 | TEA009777.1 |
|  | *GDH (GLUTAMATE DEHYDROGENASE)* | TEA031206.1 | TEA009809.1 |

**Supplemental Table 2.** Statistics table for the various phylogenetic trees.

| **Type of phylogenetic trees** | | **Number of phylogenetic trees** |
| --- | --- | --- |
| Phylogenetic trees of tea plant, rhododendron and kiwifruit | type Ⅰ | 1021 |
|  | type Ⅱ | 471 |
|  | others | 1306 |
| Phylogenetic trees of tea plant and persimmon | type Ⅲ | 67 |
|  | type Ⅳ | 10 |
|  | others | 168 |

**Supplemental Table 3.** The BLAST results for persimmon and rhododendron.

| **Pathway** | **Gene name** | **Gene ID** | **Blastp result of persimmmon** | **Blastp result of rhododendron** |
| --- | --- | --- | --- | --- |
| caffeine | *NMT* | TEA031962.1 TEA032424.1 | Dlo_pri0025F.1_g02240.1 | RHSIM_Rhsim08G0120000 |
| catechins | *SCPL 1A* | TEA034055.1 TEA023451.1 |  | RHSIM_Rhsim05G0091800 RHSIM_Rhsim05G0091900 |
|  | *PAL* | TEA014056.1 TEA023243.1 TEA003374.1 TEA003137.1 | Dlo_pri0253F.1_g01260.1 Dlo_pri0030F.1_g02210.1 | RHSIM_Rhsim13G0096300 |
|  |  |  |  |  |
|  | *ANR* | TEA022960.1 TEA009266.1 |  | RHSIM_Rhsim01G0148500 RHSIM_Rhsim08G0119200 |
|  | *ANS* | TEA015769.1 TEA010322.1 |  |  |
|  | *LAR* | TEA027582.1 TEA026458.1 |  | RHSIM_Rhsim09G0112700 RHSIM_Rhsim12G0081500 |
|  | *FLS* | TEA010328.1 TEA016601.1 |  |  |
|  | *CHS* | TEA023331.1 TEA034042.1 |  |  |
| theanine | *GOGAT* | TEA003892.1 TEA026779.1 |  | RHSIM_Rhsim03G0207200 RHSIM_Rhsim09G0205100 |
|  | *GS* | TEA032123.1 TEA032217.1 |  |  |
|  | *ADC* | TEA032991.1 TEA009777.1 |  |  |
|  | *GDH* | TEA031206.1 TEA009809.1 |  |  |


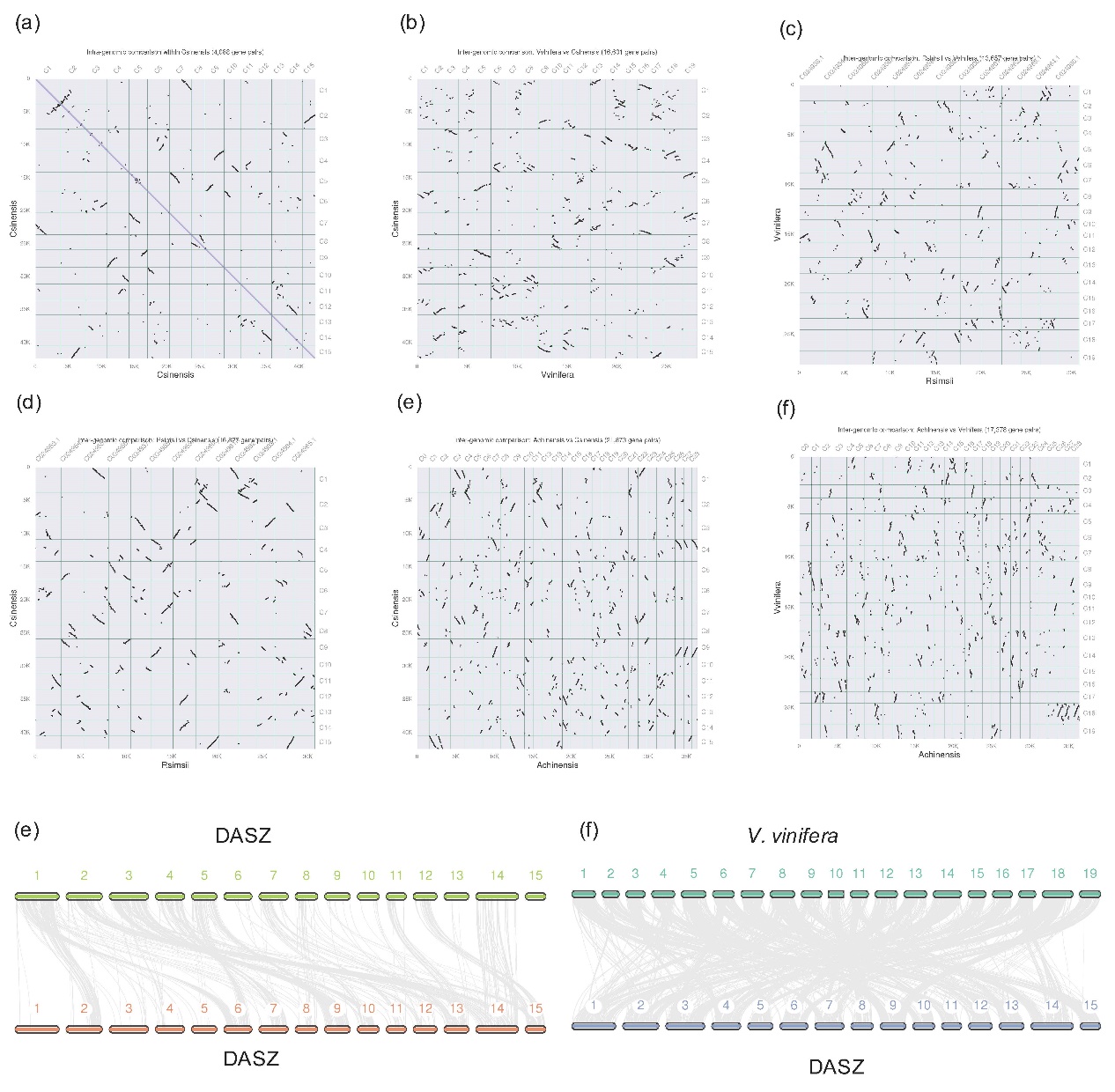


**Supplemental Figure 1.** Genomic syntenic relationships within and between tea plant (*C. sinensis*), kiwifruit (*A. chinensis*), rhododendron (*R. simsii*), persimmon (*D. lotus*) wild tea tree DASZ and grape (*V. vinifera*).

**Supplemental Figure 2.** The original *K*s distribution of tea plant (*Camellia sinensis*), kiwifruit (*Actinidia chinensis*), rhododendron (*Rhododendron simsii*) and grape (*Vitis vinifera*) with bins = 1600.


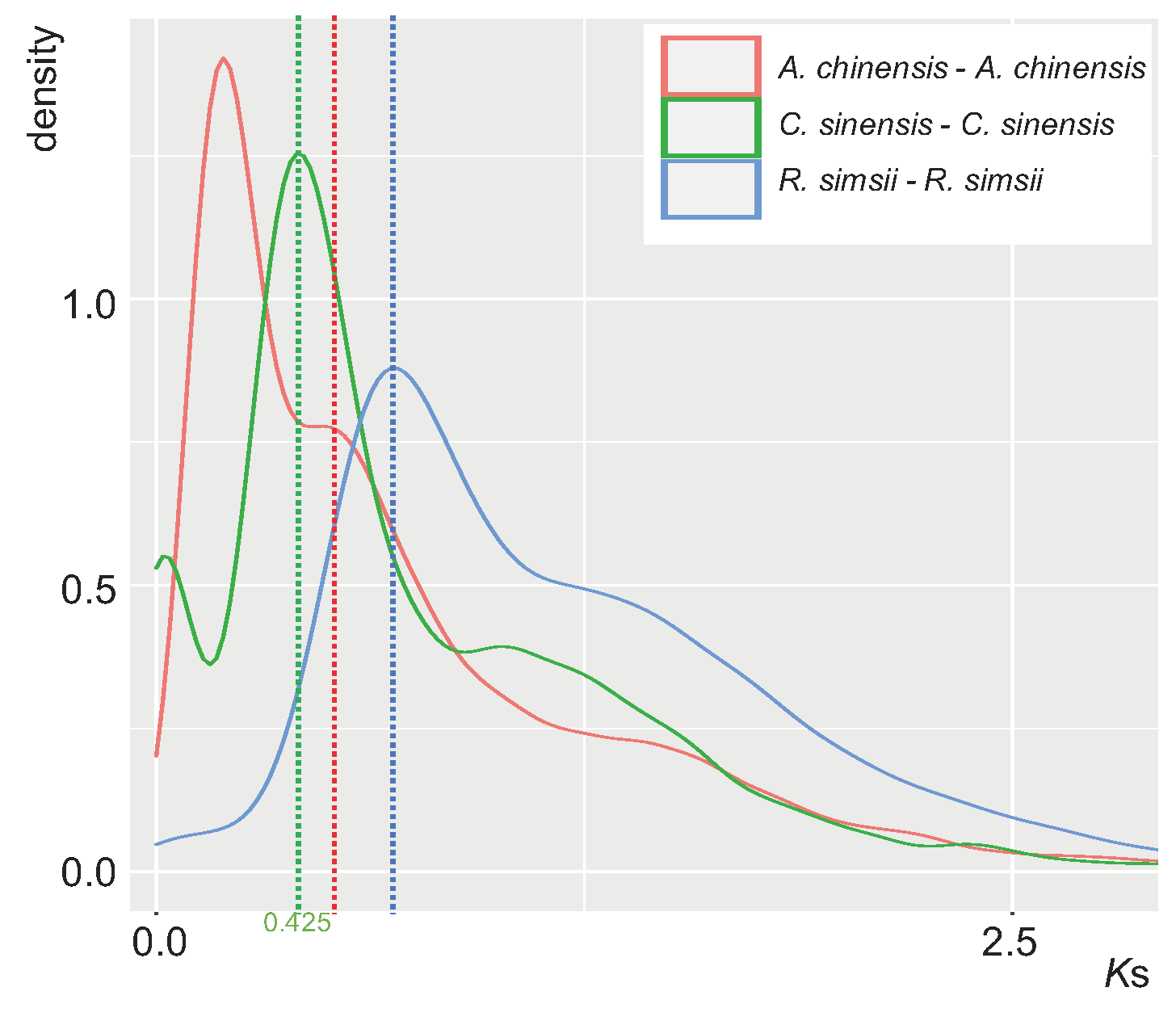


**Supplemental Figure 3.** The original unfixed *K*s distribution of tea plant (*C. sinensis*), kiwifruit (*A. chinensis*), and rhododendron (*R. simsii*).


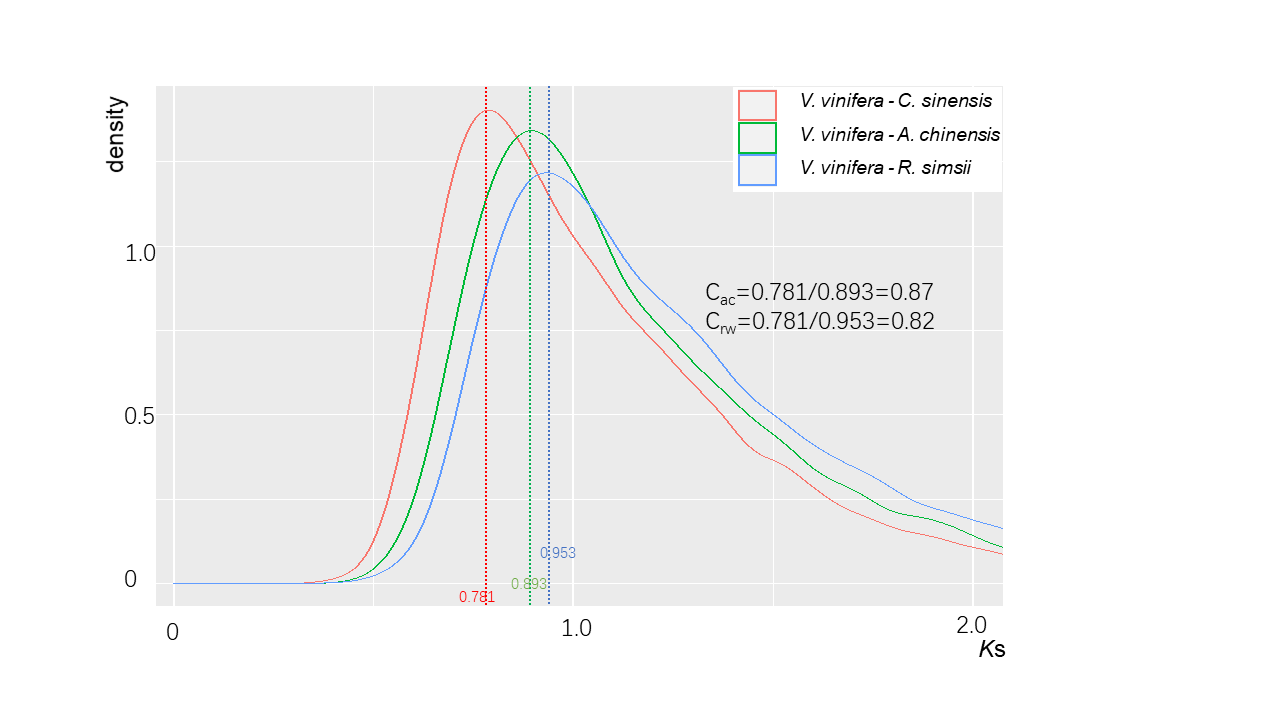


**Supplemental Figure 4.** *K*s distribution between grape (*V. vitis*) and tea plant (*C. sinensis*), kiwifruit (*A. chinensis*), and rhododendron (*R. simsii*).

**Supplemental Figure 5.** Genes involved in the biosynthesis of theanine, catechins, and caffeine are generally slightly more abundant in the tea plant than in coffee and cocoa.


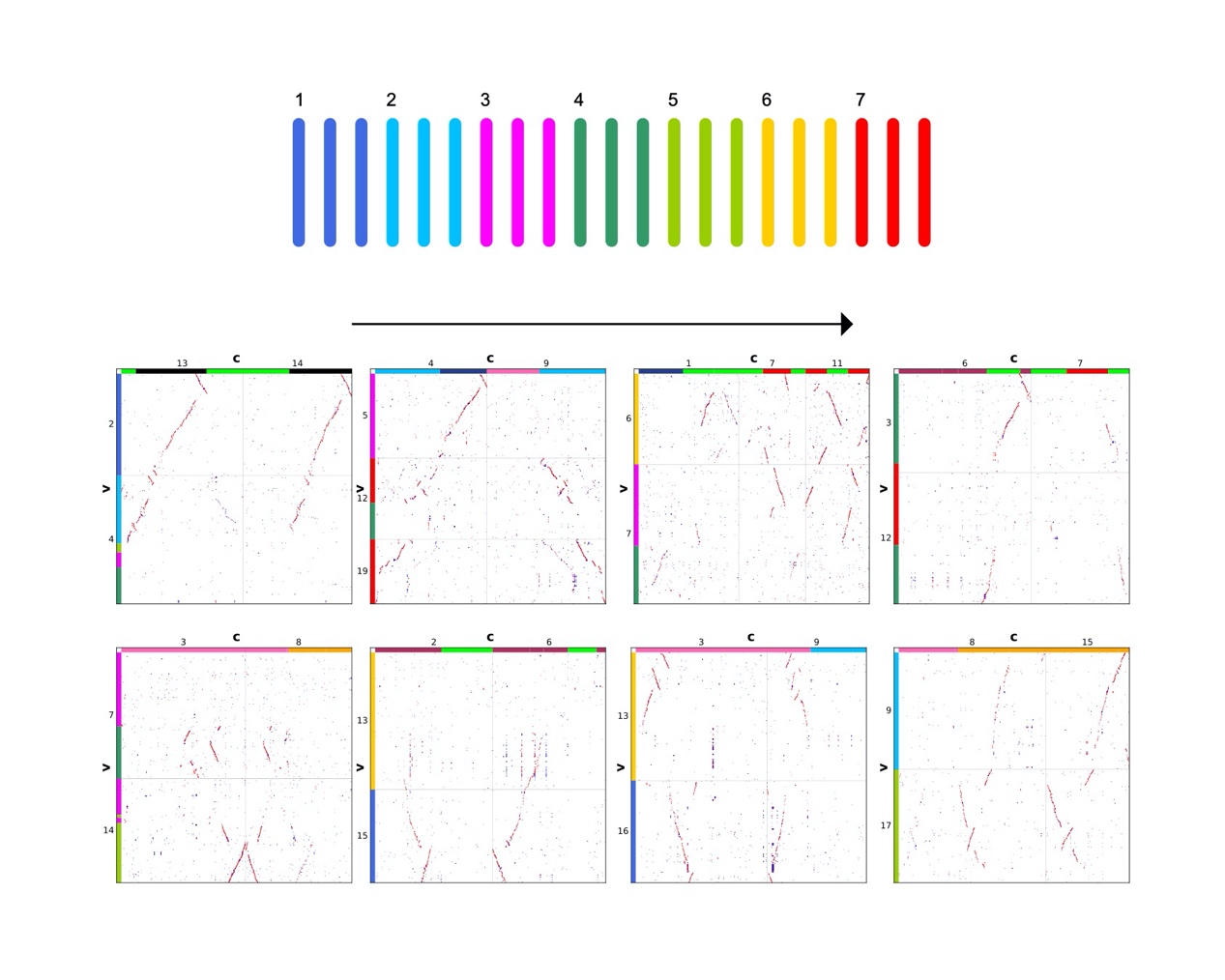


**Supplemental Figure 6.** Evolution of tea plant chromosomes from WGT to WGD. C represents tea plant (*Camellia sinensis*), V represents grape (*Vitis vinifera*).


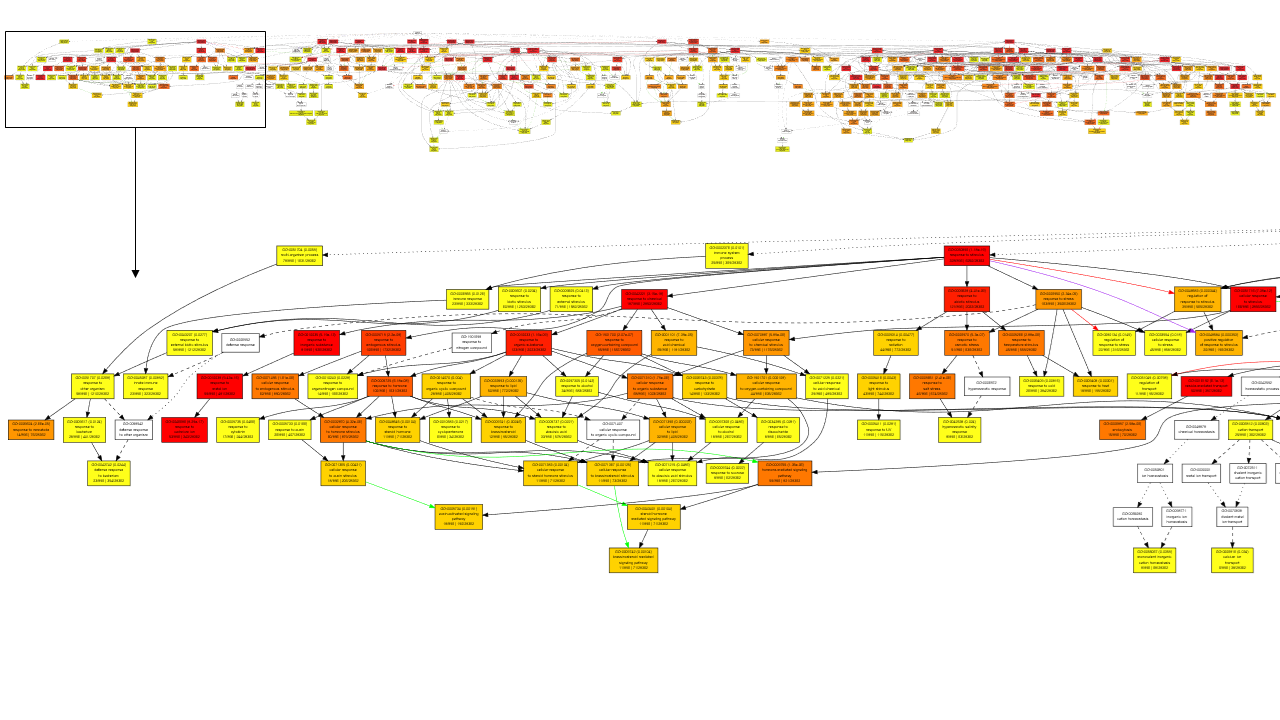


**Supplemental Figure 7.** Gene ontology analysis of WGD gene pairs in tea plant.


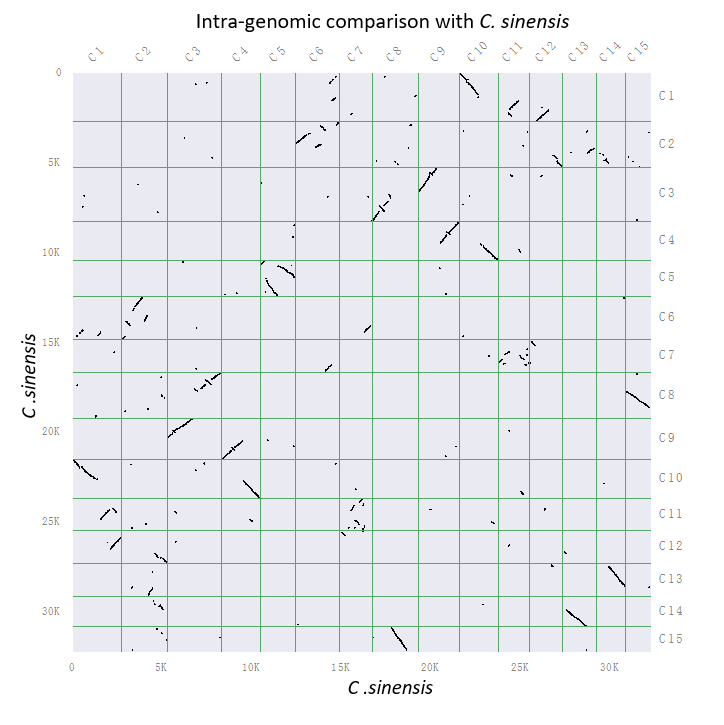


**Supplemental Figure 8.** Homologous dot plot for tea plant.
